# Construction and validation of a survival prognostic model for stage III hepatocellular carcinoma: a real-world, multicenter clinical study

**DOI:** 10.1186/s12876-023-02820-5

**Published:** 2023-06-13

**Authors:** Shuai Hao, Rongkun Luo, Wei Li, Ruhan Zhao, Tong Qi, Zichen Wang, Nan Li, Ming Liu

**Affiliations:** 1 Department of Oncology, The Third Hospital of Hebei Medical University, Shijiazhuang, Hebei PR China; 2 Graduate School of Hebei Medical University, Shijiazhuang, Hunan PR China; 3 Department of Hepatobiliary and Pancreatic Surgery, Third Xiangya Hospital, Central South University, Changsha, Hunan PR China; 4 Xiangya School of Medicine, Central South University, Changsha, Hunan PR China

**Keywords:** Hepatocellular carcinoma (HCC), Nomogram, Overall survival(OS), Prognosis

## Abstract

**Objective:**

To construct a survival prediction model for patients with TNM stage III hepatocellular carcinoma (HCC) to guide the clinical diagnosis and treatment of HCC patients and improve prognosis.

**Methods:**

Based on data from patients with stage III (AJCC 7th TNM stage) recorded by the American Institute of Cancer Research from 2010 to 2013, risk factors affecting the prognosis were screened by Cox univariate and multivariate regression, line plots was constructed, and the credibility of the model was verified by Boostrap method. ROC operating curves, calibration curves and DCA clinical decision curves were used to evaluate the model, and Kaplan–Meier was used for survival analysis was used to evaluate the efficacy of the model. External survival data from patients newly diagnosed with stage III hepatocellular carcinoma during 2014–2015 were used to validate and fit the model and to optimize the model.

**Results:**

Age > 75 years vs.18-53 years [HR = 1.502; 95%CI(1.134–1.990)], stage IIIC vs. Stage IIIA [HR = 1.930; 95%CI(1.509–2.470)], lobotomy vs. non-surgery [HR = 0.295; 95%CI(0.228–0.383)], radiotherapy vs. non-radiotherapy [HR = 0.481; 95%CI(0.373–0.619)], chemotherapy vs. Non-chemotherapy [HR = 0.443; 95%CI(0.381–0.515)], positive serum AFP before treatment vs. negative [HR = 1.667; 95%CI(1.356–2.049)], the above indicators are independent prognostic factors for patients with stage III hepatocellular carcinoma, and the *P* values for the above results were less than 0.05. A joint prediction model was constructed based on age, TNM stage, whether and how to operate, whether to receive radiotherapy, whether to receive chemotherapy, pre-treatment serum AFP status and liver fibrosis score. The consistency index of the improved prognosis model was 0.725.

**Conclusions:**

The traditional TNM staging has limitations for clinical diagnosis and treatment, while the Nomogram model modified by TNM staging has good predictive efficacy and clinical significance.

## Introduction

Hepatocellular carcinoma (HCC) can be divided into hepatocellular carcinoma, intrahepatic cholangiocarcinoma and mixed cell carcinoma, among which hepatocellular carcinoma accounts for about 90%. According to the global cancer statistics report, there will be about 840,000 new cases of hepatocellular carcinoma worldwide in 2020, and about 780,000 new deaths worldwide [1]. Data from a national survey in the United States showed that in 2022, liver cancer related deaths in males and females ranked the 7th and 5th among all malignant tumors, while studies in China showed that liver cancer ranked the first among all malignant tumors in disability-adjusted years [2–7]. In 2014, a survey data released by the World Health Organization showed that the prevalence rate of hepatitis B surface antigen in the general population in China was about 5.49%, and there were about 74 million cases of hepatitis B carriers in China. The incidence and mortality rates of primary liver cancer in China are more than half of those in the world [8, 9].

In the early stage, patients with primary liver cancer may have no obvious discomfort symptoms, or may present non-specific symptoms such as nausea, discomfort in the right upper abdomen, and a feeling of swelling in the liver area. When patients have obvious symptoms such as wasting, weakness and pain, they are mostly in the middle and late stage, and some of them have lost the opportunity for surgical resection, which is considered to be the most important radical means [10]. Its occult nature leads to poor prognosis of liver cancer. However, with the rise of radiofrequency ablation, hepatic arterial chemoembolization, radiation therapy, vascular targeted therapy, and immunotherapy, the survival rate of patients with advanced liver cancer has been greatly improved.

In clinical practice, surgical treatment is the standard treatment for patients with stage I to II patients, while the treatment strategies for stage III liver cancer are different in different medical institutions. Stage III patients include T_3a_N_0_M_0_, T_3b_N_0_M_0_, and T_4_N_0_M_0_. This stage includes multiple tumors > 5 cm in diameter, invasion of the portal vein mainly belonging to branches or hepatic veins, invasion of adjacent organs other than gallbladder, etc. As a result, treatment plans are developed differently. Therefore, there is a need to construct a new prognostic model to guide individualized diagnosis and treatment that complements the TNM staging system. AJCC/UICC TNM staging is a common and classical staging system for malignant tumors, including the size of the primary tumor, lymph node metastasis and distant metastasis, which is the basis of other staging systems. This study used survival data from a multicenter, large sample of stage III patients from the American Institute for Cancer Research to construct a prognostic prediction model designed to provide a theoretical basis for individualized treatment of patients with stage III liver cancer.

## Materials and methods

### General information

**Fig. 1:**
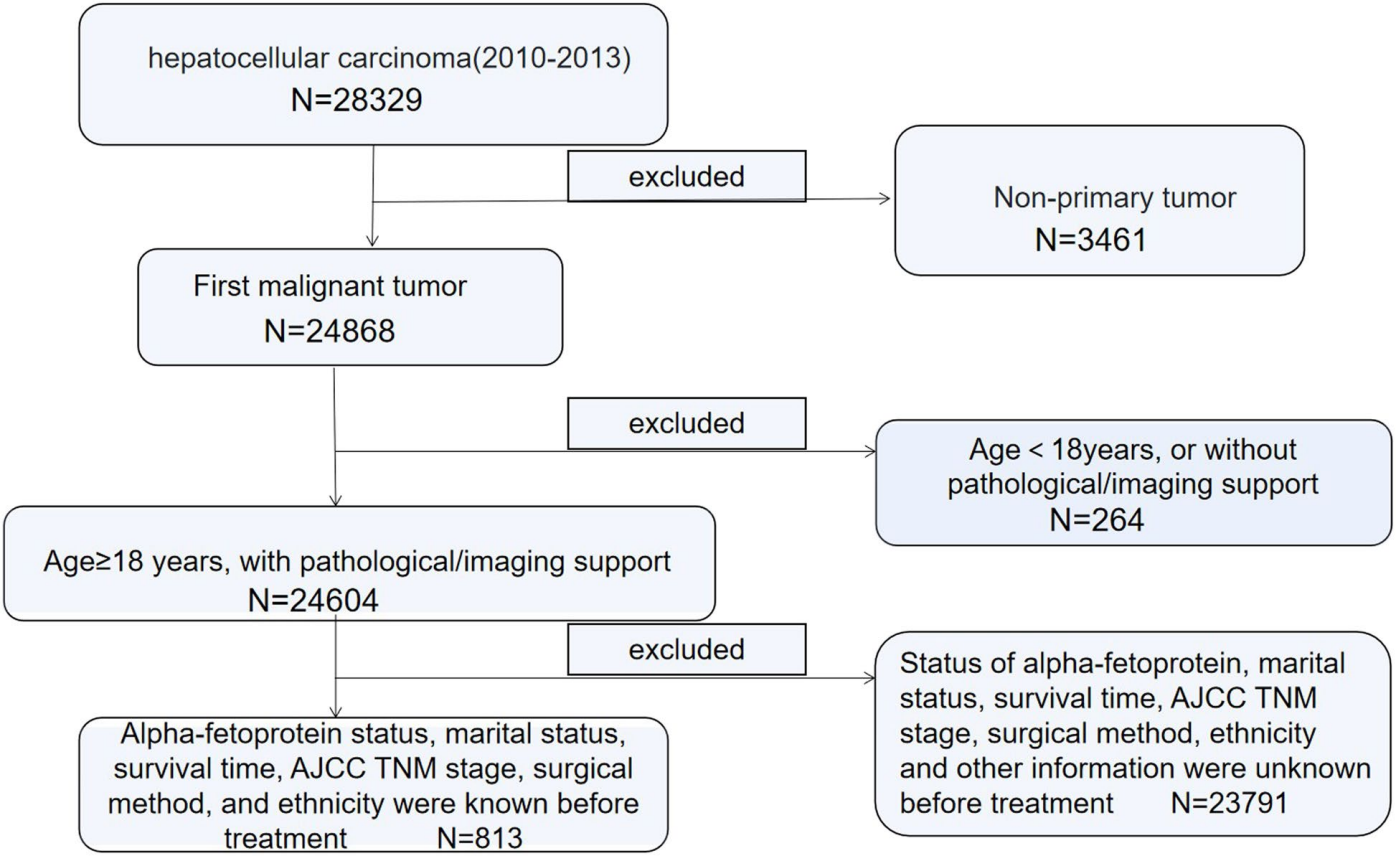
Data collection and screening flowchart

Data included were downloaded from The Surveillance, Epidemiology, and End Results Program of the National Cancer Institute. A total of 813 patients with stage III (T_3-4_N_0_M_0_) hepatocellular carcinoma (according to AJCC 7th TNM staging) with clear multi-center diagnosis were screened from 2010 to 2013. The data screening process was shown in Fig. 1. A total of 477 patients with stage III (T_3-4_N_0_M_0_) hepatocellular carcinoma diagnosed between 2014 and 2015 were collected using the same inclusion and exclusion criteria.

### Data collection

SEER*Stat 8.4.0 was used for data collection, and the collected information included: 1) Unique patient ID information; 2) Age; 3) Gender; 4) Race; 5) Diagnostic methods; 6)T stage, N stage and M stage; 7) Year of diagnosis; 8) Whether or not to undergo surgery and the surgical method; 9) Radiotherapy received or not and the type of radiotherapy; 10) Chemotherapy received or not; 11) Hepatic fibrosis score; 12) Serum alpha-fetoprotein status before treatment; 13) Marital status at the time of diagnosis; 14) Survival state and survival time; 15) Cause of death; 16) First malignant tumor; 17)WHO ICD-O-3 encoding. Inclusion criteria: a. Hepatocellular carcinoma as the only malignant tumor; b.patients diagnosed as stage III according to AJCC/UICC TNM stage 7th, including Stage IIIA, IIIB, IIIC; c.Age beyond 18 years old; d. Detailed records of surgical and chemoradiotherapy information; e. Complete follow-up information and survival time records; f. Pathological type hepatocellular carcinoma. Exclusion criteria: a. Multiple primary tumors; b. Age below 18 years old; c. Incomplete treatment information; d. Gender and marital status were unknown e. Pathological type was not hepatocellular carcinoma (bile duct cell carcinoma and mixed cell carcinoma were also excluded) f. Patients lost to follow-up and patients with incomplete survival information records.

### Validation of model

rms package, foreign package and survival package in R 4.0.4 were used to calculate the concordance index of the prognostic model. If the concordance index was greater than 0.7, the model could be considered to be highly reliable. The ROC curves of overall and tumor-specific survival at 6-month, 1-year, 2-year, and 3-year were plotted using the SurvivalROC package in R 4.0.4. The predictive value of the model increased as the area under the curves approached 1.0. The Bootstrap method was used to construct the calibration curve. The higher the degree of coincidence between the broken line and the standard line, the higher the degree of credibility of the model. DCA package was used to draw the clinical decision curve and evaluate the clinical prediction efficiency of the model.

### Processing of data

Excel 2016 was used for data cleaning, and TNM staging was summarized by referring to AJCC 7th edition hepatocellular carcinoma staging. The statistical methods involved in model construction all considered that *p* < 0.05 had statistical difference. The visualization process of data results was completed in R 4.0.4, and image integration and beautification were completed in Adobe Photograph 2020.

## Results

### Kaplan–Meier method was used to determine the Cut-off value

Kaplan–Meier analysis method in X-tile software was used to determine cut-off value of age variables. All cases were divided into three groups according to age: 18–53 years old, 54–74 years old, and ≥ 75 years old. The age fields of all cases were assigned according to the truncation value, and the continuous variables were converted into classification variables.

### Baseline clinical characteristics of modeling group and external validation group

A total of 813 patients diagnosed with stage III hepatocellular carcinoma between 2010 and 2013 were included in the modeling group, including 661 males, 152 females; 141 cases aged 18–53 years, 583 cases aged 54–74 years, 89 cases age ≥ 75 years; 680 cases were AFP positive, 133 cases were AFP negative and 664 cases had severe liver fibrosis. Between 2014 and 2015, a total of 477 stage III cases diagnosed with hepatocellular carcinoma were included in the external validation group, as shown in Table 1.

**Table 1:** Baseline characteristics of stage III hepatocellular carcinoma patients in the modeling group and the external validation group

Variable	All cases (*n* = 1290)	Training cohort (*n* = 813)	Validation cohort (*n* = 477)
Diagnose of year			
18-53 yr	193(14.96%)	141(17.34%)	52(10.90%)
54-74 yr	939(72.79%)	583(71.71%)	356(74.63%)
≥ 75 yr	158(12.25%)	89(10.95%)	69(14.47%)
Sex			
Male	1060(82.17%)	661(81.30%)	399(83.65%)
Female	230(17.83%)	152(18.70%)	78(16.35%)
Marriage status			
Single	627(48.60%)	401(49.32%)	226(47.38%)
Married	663(51.40%)	412(50.68%)	251(52.62%)
Race			
Black	153(11.86%)	96(11.81%)	57(11.95%)
White	859(66.59%)	536(65.93%)	323(67.71%)
Other	278(21.55%)	181(22.26%)	97(20.34%)
TNM			
IIIA	631( 48.91%)	394(48.46%)	237(49.69%)
IIIB	535( 41.47%)	334(25.89%)	201(15.58%)
IIIC	124( 9.61%)	85(6.59%)	39(8.18%)
AFP			
Negative	236( 18.29%)	133(16.36%)	103(21.59%)
Positive	1054( 81.71%)	680(83.64%)	374(78.41%)
Fibrosis score			
0–4	250(19.38%)	149(18.33%)	101(21.17%)
5–6	1040(80.62%)	664(81.67%)	376(78.83%)
Surgery			
No	1106(85.74%)	691(84.99%)	415(87.00%)
Local destruction	40(3.10%)	27(3.32%)	13(2.73%)
Lobe destruction	144(11.16%)	95(11.69%)	49(10.27%)
Radiotherapy			
No/Unknow	1120(86.82%)	738(90.77%)	382(80.08%)
Yes	170(13.18%)	75(9.23%)	95(19.92%)
Chemotherapy			
No/Unknow	649(50.31%)	412(50.68%)	237(49.69%)
Yes	641(49.69%)	401(49.32%)	240(50.31%)

### Cox univariate and multivariate analysis results

Age, TNM stage, alpha-fetoprotein status before treatment, surgery, radiotherapy and chemotherapy are independent prognostic factors for patients with stage III hepatocellular carcinoma (Table 2).

**Table 2:** Results of Cox univariate and multivariate analysis in patients with stage III hepatocellular carcinoma

Variable	Univariate analysis				Multivariate analysis		
	P		HR	95% CI	P	HR	95% CI
Age							
18-53 yr	Ref		1	-	Ref	1	-
54-74 yr	0.0495		1.220	1.000–1.487	0.083	1.193	0.977–1.457
≥ 75 yr	0.0045		1.492	1.132–1.966	< 0.01	1.502	1.134–1.990
Sex							
Female	Ref		1	-	Ref	1	-
Male	0.0666		0.839	0.696–1.012	Ref	1	-
Marriage							
Single	Ref		1	-	Ref	1	-
Married	0.0014		0.792	0.686–0.913	Ref	1	-
Race							
Black	Ref		1	-	Ref	1	-
White	0.213		0.868	0.695–1.085	Ref	1	-
Other	0.959		0.993	0.771–1.280	Ref	1	-
TNM							
IIIA	Ref		1	-	Ref	1	-
IIIB	< 0.001		1.700	1.459–1.981	< 0.001	1.492	1.276–1.745
IIIC	< 0.001		1.875	1.474–2.386	< 0.001	1.930	1.509–2.470
AFP							
Negative	Ref		1	-	Ref	1	-
Positive	< 0.001		1.654	1.354–2.019	< 0.001	1.667	1.356–2.049
Surgery							
No	Ref		1	-	Ref	1	-
Local	< 0.001		0.432	0.285–0.655	< 0.001	0.464	0.304–0.709
Lobe	< 0.001		0.359	0.281–0.459	< 0.001	0.295	0.228–0.383
Radiotherapy							
No/Unknow	Ref		1	-	Ref	1	-
Yes	< 0.01		0.705	0.551–0.902	< 0.001	0.481	0.373–0.619
Chemotherapy							
No/Unknow	Ref		1	-	Ref	1	-
Yes	< 0.001		0.540	0.467–0.623	< 0.001	0.443	0.381–0.515
Fibrosis score							
0–4	Ref	1		-	Ref	1	-
5–6	0.001	1.361		1.132–1.638	0.313	1.105	0.910–1.342

### Overall survival of patients with stage III hepatocellular carcinoma

**Fig. 2:**
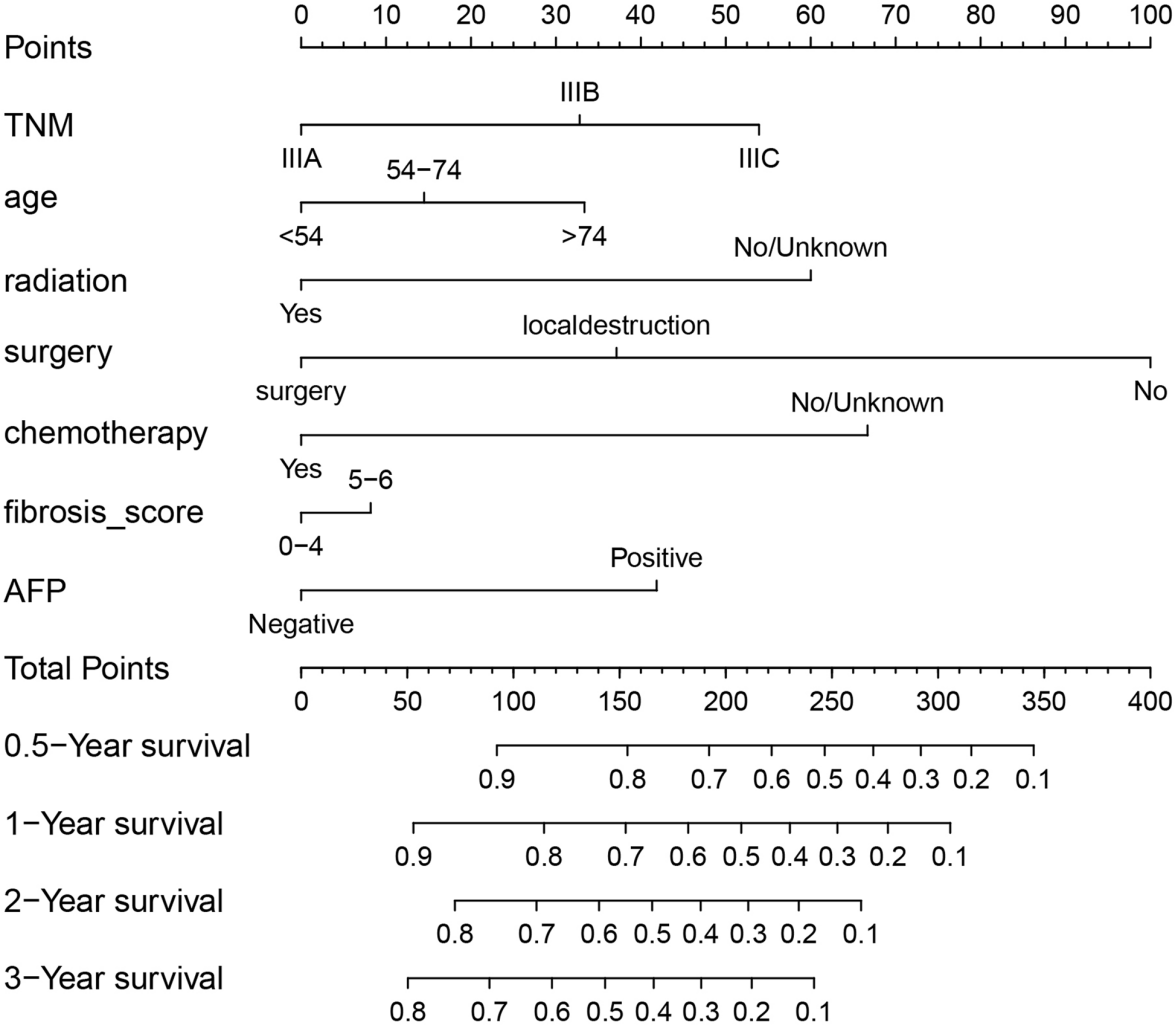
TNM staging improved prognostic nomogram model

The overall survival of patients with stage III hepatocellular carcinoma was constructed by R4.0.4 rms package, ggplot2 package and survival package. The total score of the model was 100, including the survival probability of six months, one year, two years and three years to evaluate the prognosis of patients (Fig. 2).

### Model evaluation consistency index and ROC operating curve

**Fig. 3:**
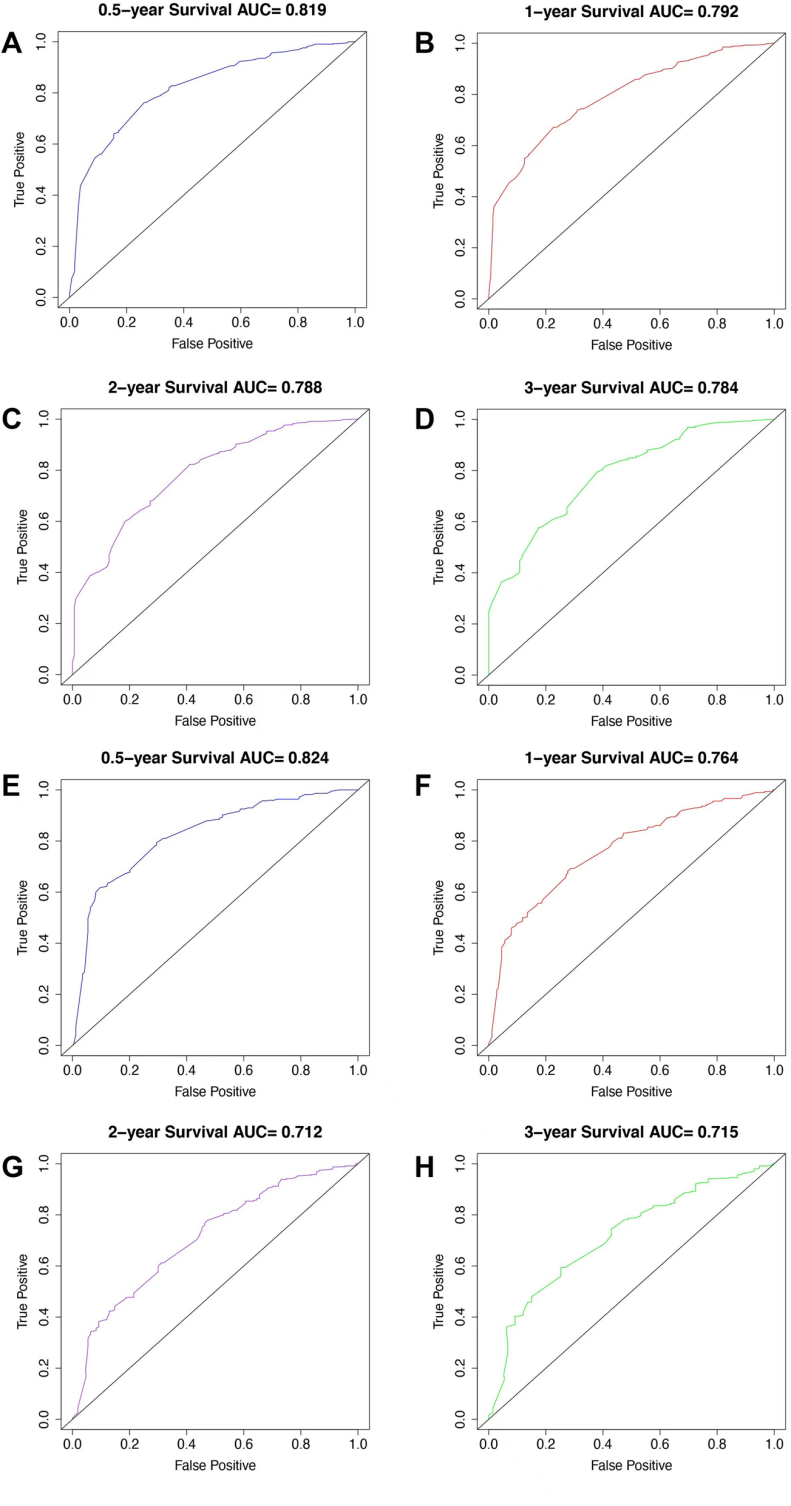
ROC operating curve evaluated the prediction results of the model (**A**-**D** modeling group 6-month, 1-year, 2-year, 3-year model prediction ability; **E**–**H** Verification of model prediction ability for 6-month, 1-year, 2-year and 3-year)

R 4.0.4 was used to calculate the reliability of the prognosis model, and the concordance index equals 0.725, indicating good reliability. The ROC test author curve was used to evaluate the 6-month, 1-year, 2-year and 3-year survival prediction results of patients in the modeling group, and the area under the curve AUC = 0.819, 0.792, 0.788, 0.784, respectively. AUC of external verification group = 0.824, 0.764, 0.712, 0.715, respectively. Figure 3A-H.

### Calibration chart to evaluate model effectiveness

**Fig. 4:**
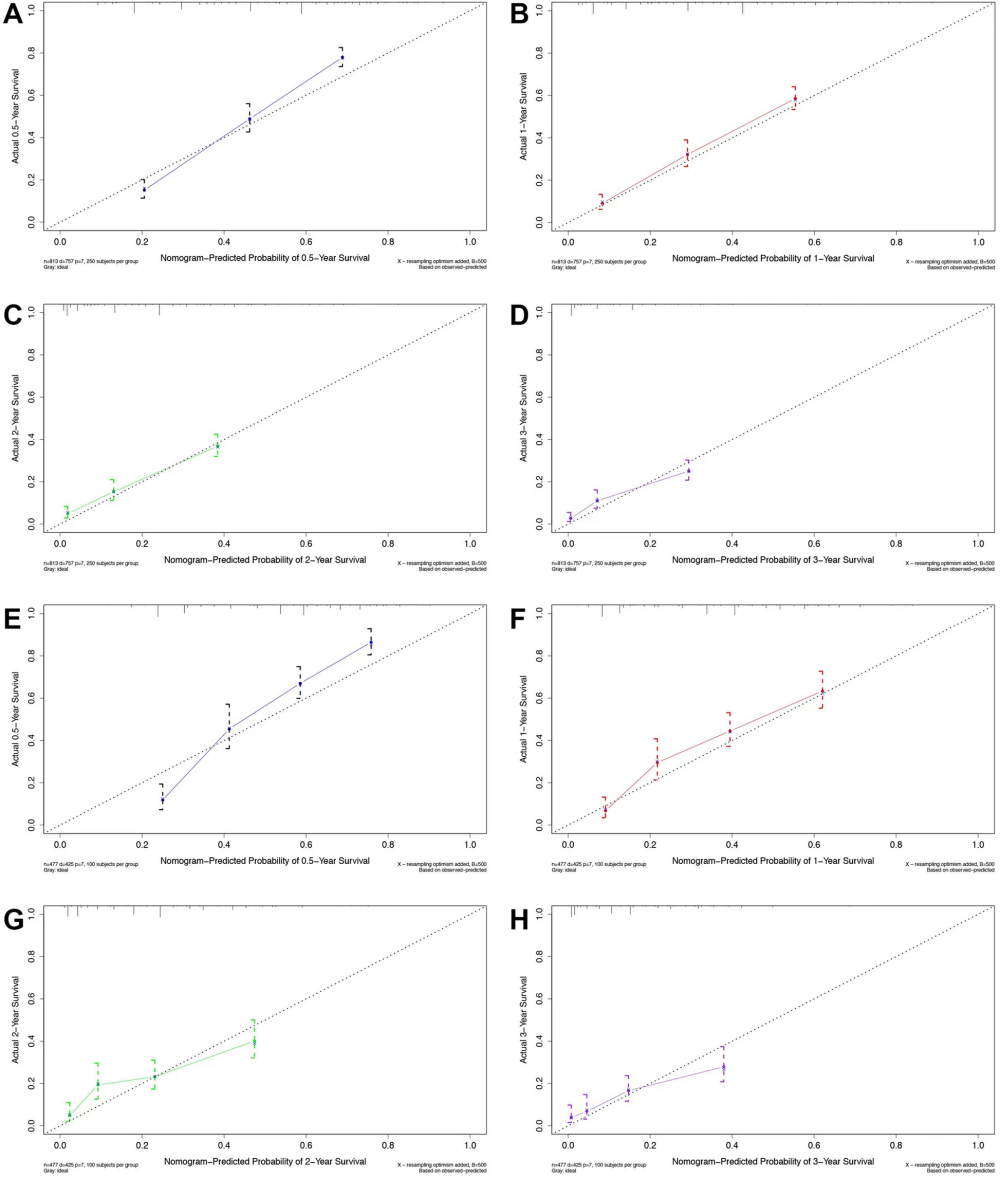
Calibration curves of the modeling group and external validation group (**A-D ** results of the modeling group at 6-month, 1-year, 2-year and 3-year; **E–H**.Results of verification group for 6-month, 1-year, 2-year and 3-year)

Calibration graph is used to assess the predictive efficiency of the model. Calibration charts were plotted for 6-month, 1-year, 2-year and 3-year respectively, as shown in Fig. 4. The curves are well aligned and the model has good predictive effeciency.

### Survival analysis by Kaplan–Meier

**Fig. 5:**
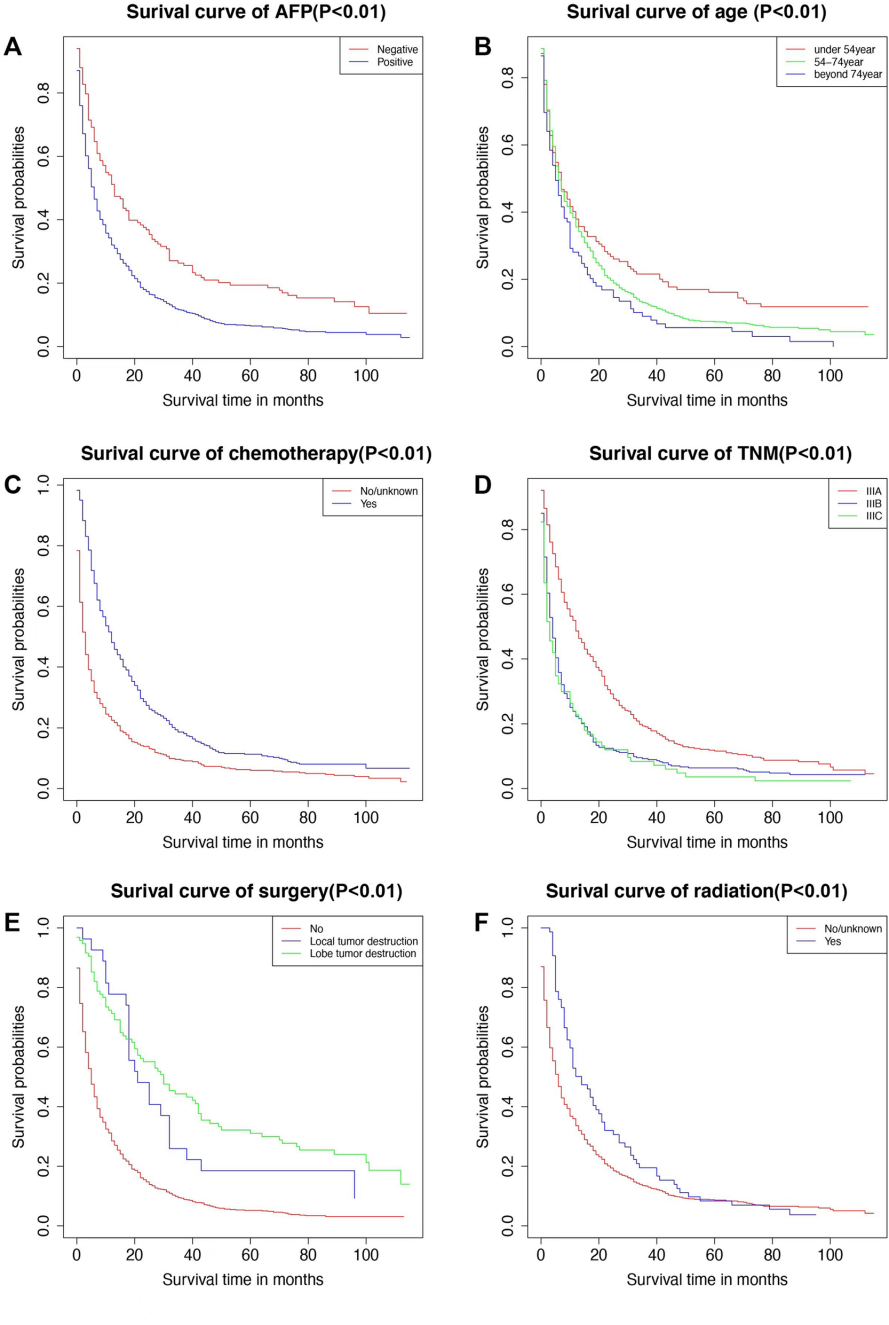
Kaplan–Meier univariate survival analysis (**A**. Serum AFP status before treatment **B**. Surgery **C**. Radiotherapy **D**.TNM stage **E**. Age **F**. Chemotherapy)

Univariate survival analysis was performed by Kaplan–Meier method, and the results were statistically different by Chi Square test with P < 0.05. The results showed that age, the status of alpha fetoprotein(AFP) before treatment, AJCC TNM stage, whether or not to receive chemotherapy, whether or not to receive radiotherapy, whether or not to have surgery and the procedure were independent risk factors for the prognosis of patients with imaging stage III (Fig. 5). All procedures were completed by R4.0.4.

### Clinical decision curve

**Fig. 6:**
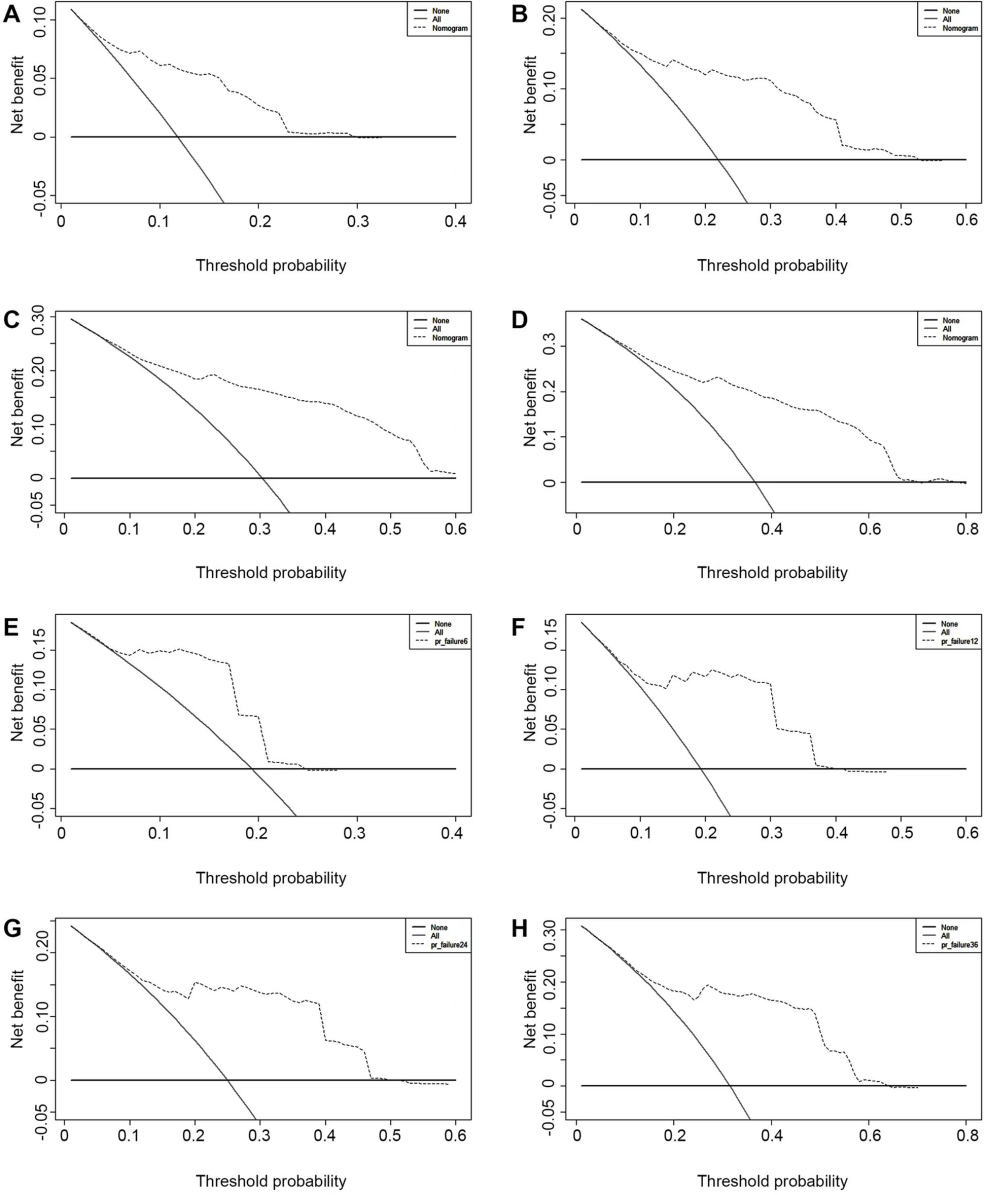
Predictive efficacy results of clinical decision curve analysis model (**A**-**D** modeling group clinical predictive efficacy evaluation **E**–**H** external validation group clinical efficacy evaluation)

The clinical decision curves for patients with stage III hepatocellular carcinoma at 6-month, 1-year, 2-year and 3-year were plotted using the R 4.0.4 DCA package, all of which demonstrated the good potential of the model for clinical application, as shown in Fig. 6A-H.

## Discussion

The etiological mechanism of liver cancer is complex and may be related to alcohol consumption, smoking, and drug abuse, and the etiology is not yet clear. However, several studies have shown that chronic hepatitis virus infection is one of the causes of liver cancer. Chronic viral infection is an important cause of hepatocellular carcinoma, and hepatitis B virus(HBV) is the most common in Asian countries, while hepatitis C virus(HCV) is the most common in Western countries. Replication of HBV or HCV viruses can induce immunosuppression. It has been suggested that uninfected patients may have longer survival compared to those infected with the chronically infected virus after treatment of Lenvatinib [11]. Data from the American Institute for Cancer Research show that the survival rate for HCC patients in the United States is 19.6 percent, and as low as 2.5 percent for those with advanced HCC. Chronic inflammation is an important cause of liver fibrosis, which is also one of the reasons for the three-step course of “hepatitis, cirrhosis and liver cancer”. In a large-scale study, it was found that 80–90% of liver cancer patients were accompanied by cirrhosis [12–14].

The present investigation involved the inclusion of various factors such as age, radiotherapy status, surgical intervention and technique, chemotherapy receipt, degree of liver fibrosis, AFP status prior to treatment, as well as TNM staging indices, in the development of a predictive model aimed at forecasting survival rates at the 6-month, 1-year, 2-year, and 3-year mark.

Upon evaluation, it was found that this model exhibits a high level of clinical prediction efficacy and has the potential to be widely implemented in clinical settings. These findings are in line with those of a large retrospective clinical study conducted by Zhang, which examined 6,603 patients and took into account factors such as age, tumor size, radiation therapy, chemotherapy, surgery, AFP, fibrosis score, and metastasis.

The study evaluated the prognosis of patients with stage IV liver cancer. The concordance index of stage IVA and stage IVB was 0.820 and 0.785 [15]. Chen also included age, gender and other factors in the model for predicting patients with hepatocellular carcinoma with severe liver fibrosis, which overcame the limitations of AJCC TNM staging. This model was highly reliable, with a consistency index of 0.781 in the training cohort and 0.793 in the verification cohort. The model is a new approach that can be extended to clinical practice and guide individualized therapy [7].

Radiotherapy can benefit the survival of patients with early, middle and advanced liver cancer, but some international guidelines do not recommend radiotherapy as a routine treatment for liver cancer. It should be pointed out that liver cancer is a radiosensitive tumor [9], but the α/β ratio of normal liver tissue is 1-2 Gy. According to the calculation of LQ model, the damage of normal liver tissue caused by large segmentation radiotherapy is greater than that caused by conventional segmentation [16]. Although stereotactic radiotherapy and adaptive radiotherapy are gradually developed in the treatment of liver cancer, However, more clinical data are needed to confirm the adverse effects of radiation induced liver injury. The study of Li pointed out that transhepatic arterial chemical perfusion combined with local radiotherapy is safe and effective [17]. A clinical meta-analysis by Ya showed that TACE combined with radiotherapy was superior to TACE alone [17, 18]. However, Wang's study pointed out that increasing the fractional dose may cause radioactive liver injury, which is independent of the total dose [16]. Nonetheless, Wang's investigation on the topic of massive liver cancer has demonstrated that radiotherapy can significantly enhance patient survival rates. Furthermore, the predictive model developed by Wang exhibited favorable clinical and practical efficacy, a finding that aligns with the conclusions drawn in the current research [9]. Ryoko’s and Jordi’s studies have confirmed that radiotherapy is an effective treatment for patients who have not received surgery or chemotherapy [19–21]. According to the results of this investigation, the implementation of radiotherapy as a supplementary treatment and protective measure for individuals diagnosed with stage III cancer can significantly enhance the overall survival rate of patients. These findings have important implications for clinical practice and underscore the significance of radiotherapy as a treatment modality.

AFP was discovered in the 1960s and was the first biomarker used in the diagnosis of hepatocellular carcinoma [22]. Chen Tian-ke’s team confirmed that AFP promoted the proliferation of hepatocellular carcinoma by inhibiting the HUR-mediated Fas/FADD apoptosis signaling pathway [23]. AFP is activated in most patients with hepatocellular carcinoma, which also predicts a poor prognosis in these patients and is also a risk factor for recurrence after treatment [24]. AFP is positive in about 70% of patients with hepatocellular carcinoma, and negative in about 30% of patients with hepatocellular carcinoma. The survival time of negative AFP patients receiving chemotherapy is longer than that of positive AFP patients [19]. The current investigation addressed the issue of serum AFP status before treatment, which was not incorporated in the TNM stage alone. Inclusion of this factor in the nomogram helped to compensate for this limitation. Analysis revealed notable differences in prognoses between patients with positive and negative AFP status, highlighting the importance of considering AFP status in treatment decision-making moving forward.A separate investigation on this topic revealed distinct independent risk factors for patients with negative versus positive AFP status, with significant differences observed in survival models as well. The findings from this study will be presented in a forthcoming publication.

Lin’s research results showed that microvascular infiltration in patients with hepatocellular carcinoma was negatively correlated with liver fibrosis, which was an independent predictor of microvascular infiltration. However, this index was not a strong prognostic factor and was not closely correlated with survival outcomes [25].

The present research found that the degree of liver fibrosis has a modest impact on prognosis, albeit with a negative correlation to survival rates. While this factor does not serve as an independent prognostic variable, it can enhance the predictive efficacy of other indicators when used in combination.

Currently, systemic therapy is widely recognized across the globe as a means of enhancing the prognosis of individuals diagnosed with hepatocellular carcinoma. In this regard, tyrosine kinase inhibitors were the initial class of drugs approved for the management of advanced-stage hepatocellular carcinoma as part of systemic treatment protocols. These inhibitors, such as regorafenib, sorafenib, and lenvatinib, are commonly utilized in conjunction with immune checkpoint inhibitors in clinical practice.The initial utilization of sorafenib as a tyrosine kinase inhibitor for hepatocellular carcinoma was later surpassed in effectiveness by lenvatinib. Nonetheless, all of these drugs—including sorafenib, regorafenib, and lenvatinib—continue to be widely employed in clinical practice. [11]. The emergence of immune checkpoint inhibitors has led to the implementation of numerous clinical trials, some of which were unsuccessful while others yielded positive outcomes for the management of hepatocellular carcinoma. However, the current use of immune checkpoint inhibitors is somewhat limited due to the occurrence of severe adverse reactions, which hinder the discontinuation of cancer treatment in favor of addressing such reactions, thereby contributing to disease progression. [26]. While various guidelines and expert consensus advocate for systemic chemotherapy with FOLFOX regimen, it is not the preferred treatment option. We observed the presence of the System Therapy field in the SEER database, but it did not specify the particular system utilized by the patient. To maintain the accuracy of our manuscript, we excluded this field. Nonetheless, this may have resulted in some degree of selection bias.

Another important factor affecting the prognosis of patients with primary hepatocellular carcinoma is liver function reserve. A large number of studies have shown that liver function is an important reference for the selection of treatment options. With the emergence of Child–Pugh classification and Meld scoring system, the evaluation system of liver function has been gradually improved, providing an important basis for clinicians to assess the liver function reserve of patients and the development of treatment options.

This study includes the index of liver fibrosis degree, which can to some extent reflect liver function. However, relying solely on this data is not ideal [27]. Regrettably, as there were discrepancies in the instruments and scoring systems used across centers, no pertinent indicators for liver function assessment were incorporated in this research. To address this issue, we intend to optimize and supplement the model using data from our own center.

While the column diagram developed in this study can help address some of the limitations of the AJCC TNM staging system, there is still room for improvement in the variables included. Additional variables, such as Child–Pugh grade of liver function [28], aminotransferase level [29], nutritional status [30], viral infection, alcohol consumption, and other indicators have been shown to be significantly associated with the prognosis of patients with primary hepatocellular carcinoma [31]. The study was a retrospective case analysis conducted in multiple centers, and these parameters were not included in the data collection. Incorporating these parameters may enhance the predictive accuracy and practicality of the model.

At present, there are more than 20 staging systems for hepatocellular carcinoma in the world. Choosing the most accurate staging system is crucial for accurate treatment. A study involving 196 patients with viral hepatitis B compared prognostic ability in patients with unresectable primary hepatocellular carcinoma and found that, Chinese University Prognostic Index(CUPI) staging system was the most suitable for predicting the prognosis and survival of patients, followed by Barcelona Clinic Liver Staging System(BCLC). Cancer of the Liver Italian Program(CLIP), Japan Integrated Staging(JIS),China integrated score(CIS), and TNM sixth edition showed poor prognostic ability [32]. The BCLC staging system is widely accepted. However, there are still defects in Barcelona Clinic Liver Staging System (BCLC). In clinical work, we were surprised to find that BCLC is mostly based on evidence from European and American populations, and the results may not be fully applicable to Asian populations. The staging system ignores some proven important methods in the treatment of hepatocellular carcinoma, such as traditional Chinese medicine and radiotherapy. The staging system is too strict for surgical resection, liver transplantation and other treatment means, so that many suitable patients lose the opportunity of radical treatment. In this study, primary tumor, lymph node metastasis, distant metastasis, treatment methods, AFP level before treatment, and degree of cirrhosis were included, all of which were reflected in BCLC, but bilirubin level, international standardized ratio and other indicators for end-stage liver disease were not included. Patients with stage III hepatocellular carcinoma were included in this study. These patients often do not develop to the end-stage of liver disease, and the above indicators have not been evaluated in the study. If the above indicators are included, the results of the study may be more accurate and the clinical application efficiency may be improved.

In the future, we plan to incorporate additional indicators related to survival, balance the baseline using propensity scores, and conduct subgroup analyses to optimize the model using visual methods. These efforts aim to enhance the clinical practicality and prediction accuracy of the model.

## Conclusion

Advanced primary hepatocellular carcinoma requires systemic therapy based on tyrosine kinase inhibitors combined with immunotherapy, supplemented by local radiation therapy can significantly improve the prognosis of patients. The choice of treatment depends on the patient 's hepatic functional reserve. The traditional TNM staging has certain limitations for clinical diagnosis and treatment, and the Nomogram model modified by TNM staging has good predictive efficacy and clinical significance.

## Data Availability

The corresponding author can be contacted to request the datasets used and analyzed during the current study, which were downloaded from https://seer.cancer.gov/. While some of the data used to support the findings of this study are included in the article, other data can also be made available upon request from the corresponding author.
